# Saturated Fatty Acid-Induced Endoplasmic Reticulum Stress and Insulin Resistance Are Prevented by Imoxin in C2C12 Myotubes

**DOI:** 10.3389/fphys.2022.842819

**Published:** 2022-07-22

**Authors:** Hyeyoon Eo, Rudy J Valentine

**Affiliations:** ^1^ Department of Kinesiology, Iowa State University, Ames, IA, United States; ^2^ Interdepartmental Graduate Program in Nutritional Sciences, Iowa State University, Ames, IA, United States

**Keywords:** imoxin, PKR, palmitate, ER stress, insulin signaling

## Abstract

In obesity, plasma free fatty acids (FFAs) levels are elevated due to enlarged adipose tissue mass. Saturated fatty acids can induce prolonged ER stress and insulin resistance. Double-stranded RNA-dependent Protein Kinase (PKR) is activated under stress conditions in skeletal muscle. The current study aimed to investigate the effect of imoxin (IMX), a selective PKR inhibitor, on palmitate-induced ER stress and insulin resistance in C2C12 myotubes. Cells were treated with 5 μM imoxin and exposed to 0.5 mM bovine serum albumin (BSA)-conjugated PA for 24 h. A subset of cells was stimulated with 50 nM insulin for the last 15 min. Glucose uptake was monitored and protein levels involved in ER stress and insulin signaling were measured by Western blotting. Palmitate stimulated PKR phosphorylation, which was prevented by imoxin. Moreover, imoxin reduced protein levels of ER stress-related markers including glucose-regulating protein 78 (GRP78), CCAAT-enhancer-binding protein homologous protein (CHOP), activating transcription factor 6 (ATF6) and spliced X-box binding protein 1 (XBP-1s) which were induced by palmitate. Furthermore, imoxin ameliorated palmitate-induced suppression of phospho-insulin receptor beta (p-IRβ) and Akt phosphorylation in myotubes. In addition, imoxin promoted glucose uptake in response to insulin under palmitate exposure. Furthermore, imoxin reduced phospho-c-Jun N-terminal kinase (p-JNK) induced by palmitate treatment. These findings suggest that imoxin may protect against saturated fatty acid-induced ER stress and insulin resistance in skeletal muscle, which are potentially mediated by PKR.

## Introduction

Obesity is a major public health issue worldwide (De Lorenzo et al., 2019), characterized by expansion of adipose tissues due to imbalance between food intake and energy expenditure (Gomez-Hernandez et al., 2016). The expanding fat mass releases free fatty acids (FFAs), mostly in the form of the saturated fatty acid (FA) palmitic acid or palmitate, into the blood stream (Boden, 1998; Gambino et al., 2016; Carta et al., 2017). Circulating FFA levels are markedly elevated in individuals with obesity (Arner and Ryden, 2015). Many researchers have shown that infusion or administration of palmitate PA induces insulin resistance in multiple tissues (Benoit et al., 2009; Tran et al., 2016) as well as cells (Yang et al., 2013; Tran et al., 2016). In addition to insulin resistance, it was reported that FFAs can promote endoplasmic reticulum (ER) stress in various cell types (Wei et al., 2006; Cao et al., 2012; Perry et al., 2018; Sharmin et al., 2020).

The ER is a multifunctional organelle involved in lipid synthesis, protein folding and post-translational modification (Tripathi and Pandey, 2012). Mildly stressful conditions in the ER can be dealt with through the unfolded protein response or UPR (Sarvani et al., 2017). However, chronic and prolonged stress due to physiological, pathological, and pharmacological conditions can disrupt ER homeostasis and protein folding capacity in the ER, which leads to ER stress (Sicari et al., 2020). ER stress results from accumulation of unfolded or misfolded protein in the ER lumen (Kitamura, 2008; Cnop et al., 2012). Several studies have reported that ER stress inducers such as tunicamycin and thapsigargin block insulin signaling (Quan et al., 2015; Brown et al., 2020). ER stress-induced insulin resistance is mediated by activation of c-Jun N-terminal kinase (JNK) which promotes serine/threonine phosphorylation of insulin receptor substrate-1 (IRS1) resulting in IRS1 degradation (Ozcan et al., 2004). Therefore, controlling ER stress in metabolic tissues such as skeletal muscle is important for maintenance of glucose homeostasis.

In our previous research, we found that imidazole-oxindole, or imoxin treatment suppressed tunicamycin-induced ER stress and restored insulin signaling impaired by tunicamycin in C2C12 myotubes (Eo and Valentine, 2021). Imoxin, a double-stranded RNA (dsRNA) activated protein kinase (PKR) inhibitor, promoted glucose uptake suppressed by ceramide which is synthesized from saturated FA during overload of fatty acids (Hage Hassan et al., 2016). Additionally, PKR-deficiency protected insulin sensitivity from high-fat diet feeding in mice (Carvalho-Filho et al., 2012). However, it has yet to be established whether PKR is involved in the metabolic disturbances induced by saturated FA-induced ER stress and insulin resistance in skeletal muscle. In this context, the current study aimed to evaluate the protective effect of imoxin against palmitate-induced ER stress as well as impairment of glucose uptake through insulin signaling pathways in skeletal muscle cells. It was hypothesized that imoxin would suppress palmitate -induced ER stress and reinstate insulin signaling and glucose uptake in the context ER stress.

## Materials and Methods

### Cell Culture and Differentiation

Mouse C2C12 myoblasts were purchased from American Type Tissue Culture (ATCC CRL-1772). Myoblasts were cultured in high-glucose (25 mM glucose) Dulbecco’s modified Eagle’s medium (DMEM; Gibco, Waltham, MA) supplemented with 10% fetal bovine serum (Gibco, Waltham, MA), 1% penicillin-streptomycin, and 1% Glutamax in 5% CO2 at 37°C. Upon reaching 90% confluence, growth medium was replaced with differentiation medium [high-glucose DMEM containing 2% horse serum (Gibco, Waltham, MA), 1% penicillin-streptomycin, and 1% Glutamax], and replaced every other day for 5 days for cells to be fully differentiated.

### Fatty Acids Preparation and Cell Treatment

Bovine serum albumin (BSA)-conjugated palmitate was prepared by following the procedure as previously described with some modifications (Gao et al., 2004; Li et al., 2018). Briefly, a stock of 200 mM palmitate (NU-CHEK PREP, INC., Elysian, MN) in 100% EtOH was mixed with 3.3% fatty acid-free BSA solution (MilliporeSigma, Burlington, MA) to a working concentration of 1.7 mM by heating at 40°C for 1 h (the molar ratio of palmitate to BSA was 3.4). The same volume of EtOH mixed with 3.3% fatty acid-free BSA was used as control. The BSA or BSA-conjugated palmitate stock solution was then dissolved in DMEM containing 50 μM carnitine, 10 mM HEPES (pH 7.3), 26 mM NaHCO3, and 1 μg/ml gentamicin, to a final concentration of 0.5 mM palmitate (1% BSA).

After 5 days of differentiation, myotubes were treated with vehicle [VEH; phosphate buffered saline (PBS)] or 5 μM imoxin (EMD Millipore, Burlington, MA) in the absence or presence of 0.5 mM BSA-conjugated palmitate for 24 h. To evaluate insulin action, 50 nM insulin was added to the myotubes 15 min before harvest.

### Western Blot Analysis

Cells were harvested using 1X Cell Lysis Buffer (Cell Signaling Technology, Danvers, MA) containing 1X Halt™ protease inhibitor cocktail (ThermoFisher Scientific, Waltham, MA) and 1X phosphatase inhibitor cocktail (MilliporeSigma, Burlington, MA). Protein quantification was performed by using a BCA assay kit (ThermoFisher Scientific, Waltham, MA) and then 6–10 ug of protein sample were loaded on each well of 4%–15% Criterion™ TGX Stain-Free™ Protein Gel (Bio-Rad, Hercules, CA). Protein samples were separated and transferred to polyvinylidene difluoride membrane (MilliporeSigma, Burlington, MA). Gels were activated according to Bio-Rad’s Stain-Free protocol, and total protein was determined by imaging the membrane under UV light to normalize signal intensity for the proteins of interest to the total protein in the same lane. The abundances of target proteins and their phosphorylated forms were detected by western blot analysis as previously described (Eo and Valentine, 2021). The primary antibodies were purchased and directed against proteins as follows: p-PKR (Thr446, ab32036) purchased from Abcam (Cambridge, United Kingdom); PKR (sc-6282), GRP78 (sc-13539), PKR-like ER eukaryotic translation initiation factor 2 alpha kinase (PERK, sc-377400) purchased from Santa Cruz Biotechnology, Inc. (Dallas, TX); CHOP (#2895), p-PERK (#3179), activating translational factor 4 (ATF4, #11815), ATF6 (#65880), spliced X-box binding protein 1 (XBP-1s, #83418), p-insulin receptor beta (p-IRβ; Tyr1150/1151, #3024), insulin receptor substrate 1 (IRS1; #3407), p-IRS1 (Ser636/639, #2388), p-Akt (Ser437, #9271), Akt (#9272), Akt substrate of 160 kDa (AS160, #2670), p-AS160 (Thr642, #8881), and phosphorylated stress-activated protein kinase/c-Jun amino-terminal kinase (p-SAPK/JNK, Thr183/Tyr185, #4668) purchased from Cell Signaling Technology (Danvers, MA). Secondary antibodies against mouse (#7076) and rabbit (#7074) were purchased from Cell Signaling Technology. The blots were imaged using an enhanced chemiluminescence solution (Thermo Fisher Scientific, Waltham, MA). Immunoreactive bands were captured with the ChemiDoc XRS Imaging System (Bio-Rad, Hercules, CA), and densitometry was performed using Image Lab v6.0 (Bio-Rad, Hercules, CA).

### Glucose Uptake Determination

Glucose uptake was monitored by using a fluorescent D-glucose analog 2-[N-(7-nitrobenz-2-oxa1,3-diazol-4-yl)amino]-2-deoxy-D-glucose (2-NBDG, Cat# 11,046, Cayman Chemical Company, Ann Arbor, MI). Fully differentiated myotubes were treated with or without 5 μM imoxin in the absence or presence of 0.5 mM BSA-conjugated palmitate for 23 h. Cells were washed with sterile PBS and then stimulated with or without 50 nM insulin in glucose-free/serum-free DMEM for 30 min, followed by the addition of 2-NBDG at the final concentration of 100 μg/ml for an additional 30 min. The medium was then removed, and cells were washed twice with PBS and lysed with 0.5% Triton X-100 in 100°ul PBS to stop the further insulin stimulation. The fluorescent intensity of cellular 2-NBDG in each well was measured at excitation/emission wavelengths of 475 and 550 nm using a microplate reader (Fluostar Galaxy, BMG Labtech, Ortenberg, Germany).

### Statistical Analysis

**FIGURE 1:**
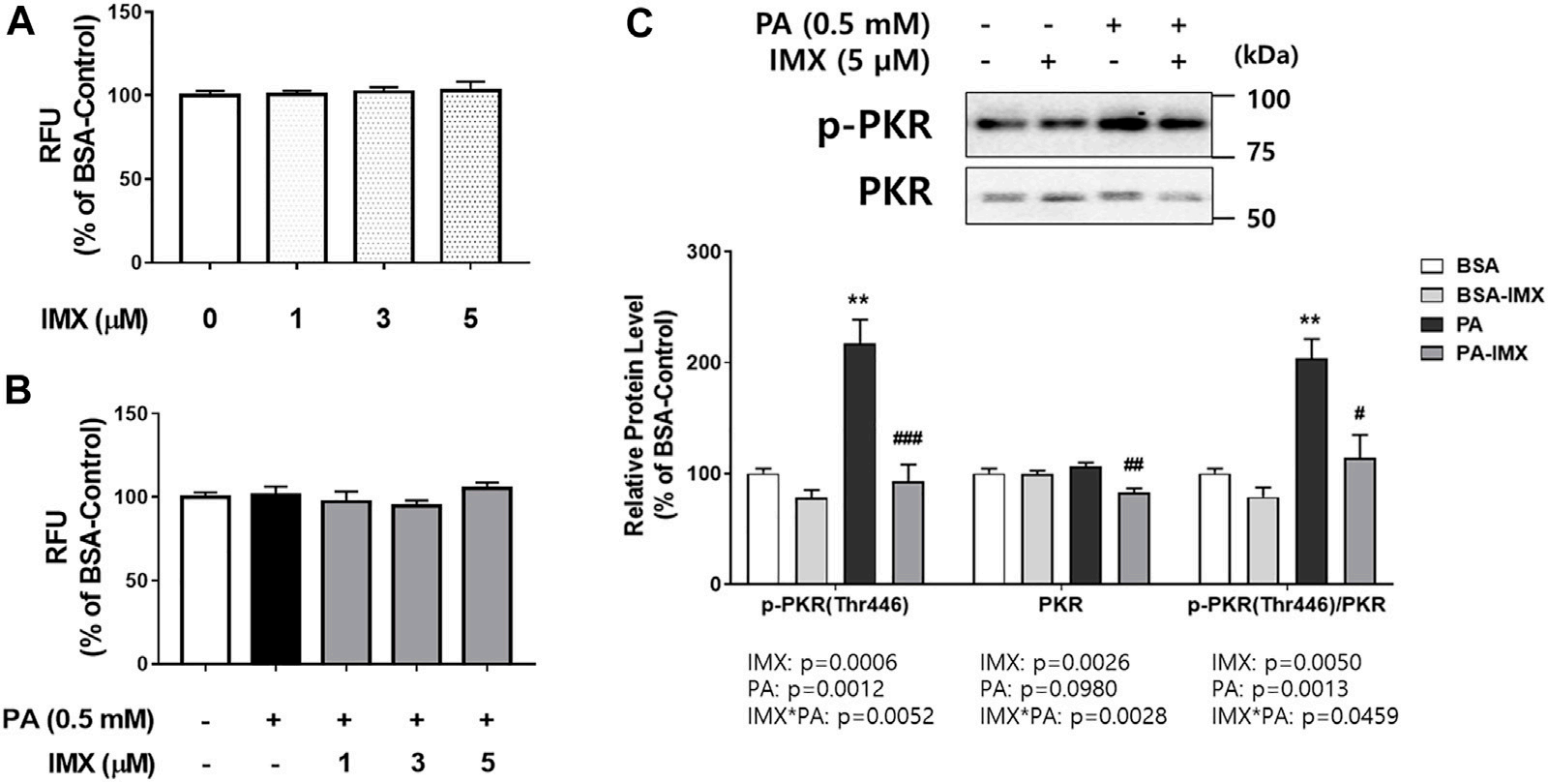
Effect of palmitate and imoxin on PKR phosphorylation in C2C12 myotubes. Cells were treated with 5 μM imoxin (IMX) with or without media containing 0.5 mM BSA-conjugated palmitate (PA) for 24 h. Cell viability was assessed **(A, B)**. Protein levels of p-PKR (Thr446) and total PKR were measured by western blot **(C)**. Representative images are shown on the top panel and quantification is shown on the bottom. Data were analyzed by two-way ANOVA (IMX x PA) followed by Tukey’s post-hoc test. Values are presented as mean ± SEM (N = 3). **p < 0.01 compared with the BSA-control group; #*p* < 0.05, ##*p* < 0.01, ###*p* < 0.001 compared with the PA-only group.

**FIGURE 2:**
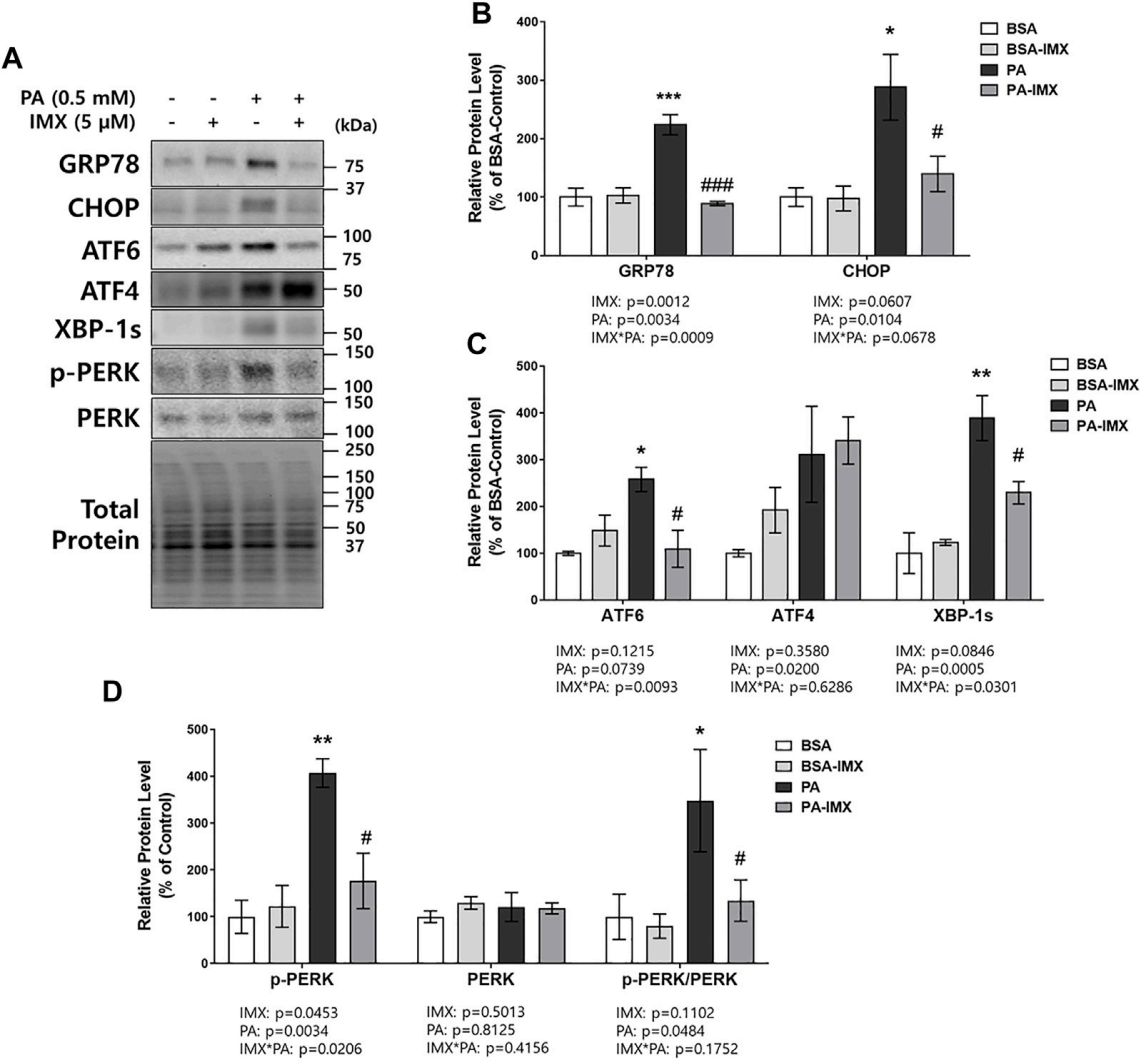
Effect of imoxin on protein levels of ER stress-related markers stimulated by palmitate in C2C12 myotubes. The cells were treated with imoxin (IMX) at 5 μM with/without 0.5 mM palmitate (PA) for 24 h. Protein level of each biomarker was measured by western blot. **(A)** Representative band images and **(B–D)** quantification data are shown. Values are presented as mean ± SEM (N = 3). Main effects and interactions were analyzed by two-way ANOVA (IMX x PA), followed by Tukey’s post-hoc test: *p < 0.05, **p < 0.01 and ***p < 0.001 compared with the BSA-control group; #*p* < 0.05 and ###*p* < 0.001 compared with the PA only group.

**FIGURE 3:**
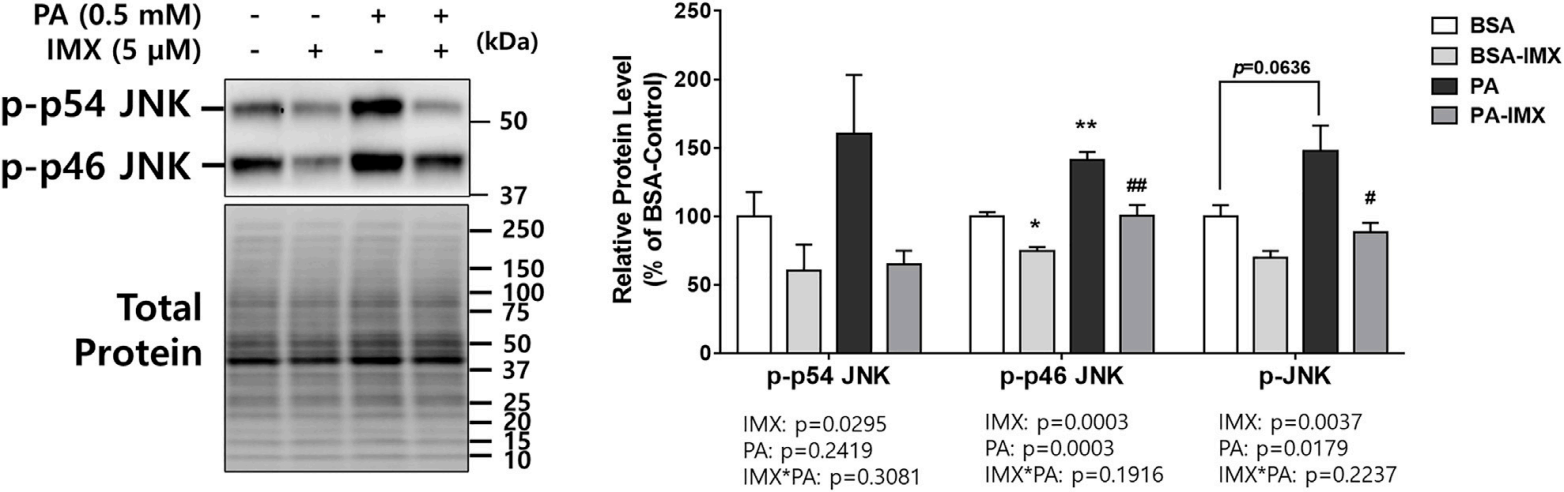
Effect of palmitate and Imoxin on p-JNK expression in C2C12 myotubes. The cells were treated with imoxin (IMX) at 5 μM with/without 0.5 mM palmitate (PA) for 24 h. Protein level of p-JNK (p-p54/p46) was measured by western blot. Representative band images are shown on the left panel and quantification of each phosphorylation site and their summation is shown on the right panel. Differences between treatments were analyzed by two-way ANOVA (IMX x PA). Values are presented as mean ± SEM (N = 3). *p < 0.05 and **p < 0.01 compared with the BSA-control group; #*p* < 0.05 and ##*p* < 0.01 compared with the PA only group.

**FIGURE 4:**
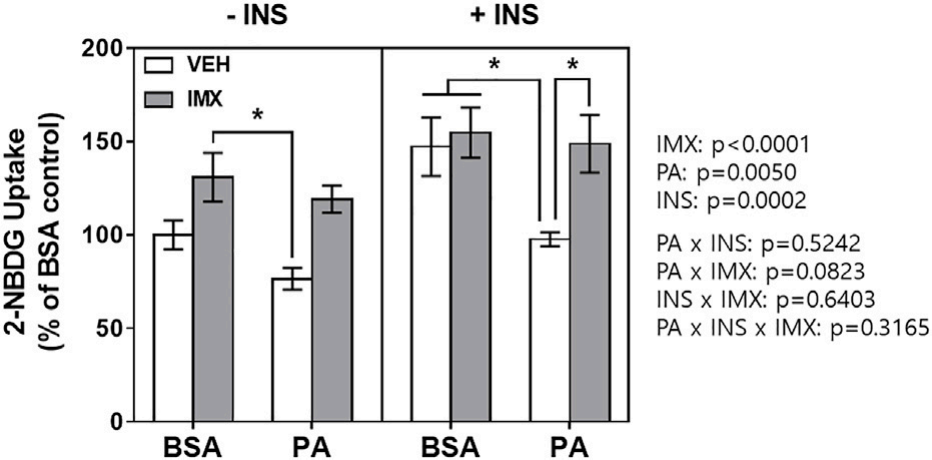
Effect of palmitate and imoxin on glucose uptake in C2C12 myotubes. Glucose uptake monitored by 2-NBDG assay. To stimulate glucose uptake, cells were washed with sterile PBS twice and then treated with or without 50 nM insulin in serum-free DMEM without glucose for 30 min. After 30 min of insulin stimulation, 2-NBDG was added to cells at the final concentration of 100 μg/ml for another 30 min. Values are presented as mean ± SEM (N = 3). Main effects and interactions were analyzed by three-way ANOVA (IMX x PA x insulin) as described on the right side of the panel: *p < 0.05 by Tukey’s post hoc analysis after three-way ANOVA.

**FIGURE 5:**
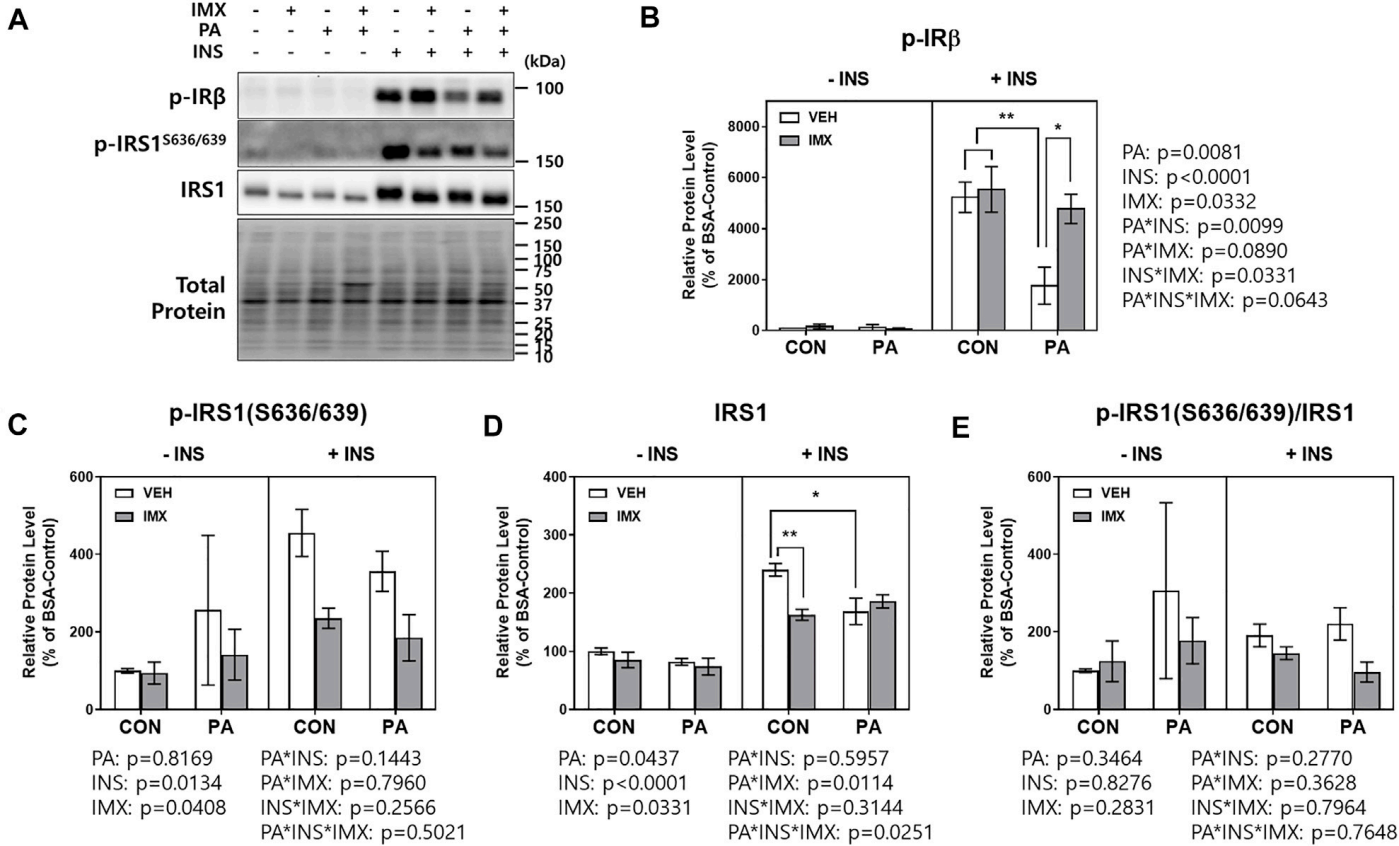
Effect of imoxin on insulin-stimulated p-IRβ and IRS1 phosphorylation in presence or absence of palmitate in C2C12 myotubes. The cells were treated with imoxin (IMX) at 5 μM with/without 0.5 mM palmitate (PA) for 24 h. Protein level of each biomarker was measured by western blot. **(A)** Representative band images and **(B–E)** quantification data of each biomarker are shown. Differences between treatments were analyzed by three-way ANOVA (IMX x PA x insulin). Values are presented as mean ± SEM (N = 3). *p < 0.05 and **p < 0.01 by Tukey’s post hoc analysis after three-way ANOVA.

**FIGURE 6:**
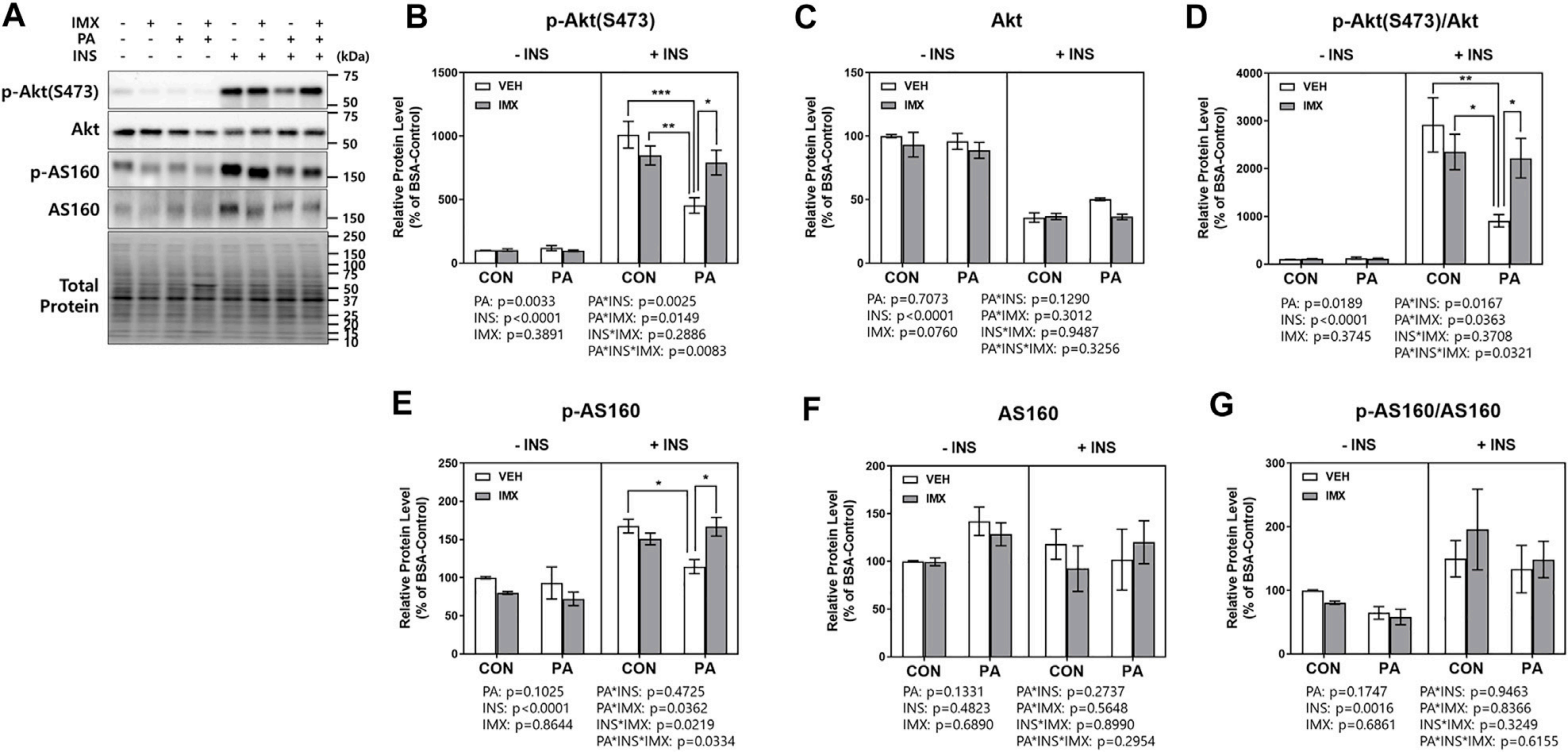
Effect of imoxin on insulin-stimulated Akt and AS160 phosphorylation in presence or absence of palmitate in C2C12 myotubes. The cells were treated with imoxin (IMX) at 5 μM with/without 0.5 mM palmitate (PA) for 24 h. Protein level of each biomarker was measured by western blot. **(A)** Representative band images and **(B–G)** quantification data of each biomarker are shown. Differences between treatments were analyzed by three-way ANOVA (IMX x PA x insulin). Values are presented as mean ± SEM (N = 3). *p < 0.05, **p < 0.01 and ***p < 0.001 by Tukey’s post hoc analysis after three-way ANOVA.

All statistical analyses were performed using GraphPad Prism 7.0.5 (GraphPad Software Inc., San Diego, CA). Data from experiments replicated 3 times [biological replicates (N = 3)] with three technical replicates (N’ = 3) each are expressed as the mean ± standard error of means (SEM) and the statistical analyses were performed using biological replicates (N = 3). A two-way ANOVA with Tukey’s post-hoc test was performed for comparisons of protein levels (Figures 1–3). A three-way ANOVA with Tukey’s post-hoc test was performed for multiple comparisons in levels of proteins involving in insulin signaling pathways (Figures 4–6). The differences between groups and the main effects of palmitate, imoxin and insulin were considered statistically significant at *p* < 0.05 and shown in each figure. Specifically, main effects are described as *p*-values within each figure when data were analyzed with two-way or three-way ANOVAs.

## Results

### Effect of Palmitate and PKR Phosphorylation in C2C12 Myotubes

The effect of palmitate and imoxin on protein levels of PKR and its phosphorylation was evaluated using western blot (Figure 1). Interactions between palmitate and imoxin were found in protein levels of p-PKR and PKR and PKR phosphorylation (p-PKR/PKR ratio). Palmitate promoted PKR phosphorylation (103% increase) at Thr446 compared with the BSA-control, even though there was no effect of palmitate on total PKR. However, imoxin decreased both protein levels of PKR and PKR phosphorylation compared with the palmitate -only treatment by 23% and 44%, respectively. According to the two-way ANOVA there were main effects of palmitate to increase p-PKR protein expression and PKR phosphorylation, and imoxin to reduce protein levels of p-PKR and total PKR as well as PKR phosphorylation (ratio of p-PKR/total PKR).

### Effect of Imoxin on Palmitate-Induced ER Stress Signaling Molecules in C2C12 Myotubes

To evaluate the effect of palmitate and imoxin on the UPR, protein levels of ER stress markers were measured in C2C12 myotubes by performing western blot assays (Figure 2). Significant increases in protein levels of ER stress markers including GRP78 (131%), CHOP (187%), ATF6 (157%) and XBP-1s (289%) were observed in the cells exposed to palmitate compared to the BSA-control group (Figures 2B,C). In comparison to palmitate alone, imoxin treatment significantly lowered protein levels of ER stress markers including GRP78 (60%), CHOP (48%), ATF6 (58%) and XBP-1s (41%) compared to palmitate alone (Figures 2B,C). Such changes represent a full prevention of palmitate-induced GRP78 and ATF6, whereas other markers were slightly less robust. A main effect of palmitate was found, with increases in protein levels of ATF4 (Figure 2C), although there were no individual differences across treatment groups. In addition, palmitate stimulated PERK phosphorylation (p-PERK/PERK; 248% increase compared with the BSA-control), which was significantly reduced by imoxin by 61% (Figure 2D).

### Effect of Imoxin and Palmitate on p-JNK Expression in C2C12 Myotubes

JNK is responsible for ER stress induced insulin resistance through promotion of serine/threonine phosphorylation of IRS1 which results in IRS1 degradation (Solinas and Becattini, 2017). For this reason, the current study evaluated protein levels of p-JNK (measuring both p-p54 and p-p46) in myotubes (Figure 3). According to the two-way ANOVA, there was a main effect of palmitate to increase p-p46 and total p-JNK. Similarly, palmitate significantly increased the protein level of p-p46 JNK (41% increase) compared to the BSA-control, but there were no differences in protein levels of p-p54 or p-JNK between palmitate and BSA control. On the other hand, there were main effects of imoxin on protein expression of p-p54 JNK, p-p46 JNK and p-JNK (Figure 3). Post-hoc tests revealed that imoxin treatment decreased protein levels of p-p46 JNK compared to control, in both the absence and presence of palmitate (by 25% and 29%, respectively), and total p-JNK in the presence of palmitate (by 40%), but did not significantly lower p-p54 JNK within either condition.

### Effect of Palmitate and Imoxin on Glucose Uptake in C2C12 Myotubes

The 2-NBDG assay was performed to measure glucose uptake in C2C12 myotubes (Figure 4). There were main effects of imoxin, palmitate, and insulin in 2-NBDG glucose uptake. Palmitate reduced 2-NBDG uptake in presence of insulin (by 34%). However, imoxin treatment significantly promoted insulin-stimulated 2-NBDG uptake (152% increase) which was suppressed by palmitate.

### Effect of Palmitate and Imoxin on Insulin Signaling in C2C12 Myotubes

To evaluate the effects of palmitate and imoxin on the insulin signaling pathway, C2C12 myotubes were treated with imoxin and BSA-conjugated palmitate as described above and then 50 nM insulin was added. There were main effects of palmitate, insulin, imoxin, palmitate*insulin and insulin*imoxin in protein levels of p-IRβ (Figure 5B). As shown in Figure 5, palmitate significantly suppressed insulin-stimulated p-IRβ compared with BSA-control, which was prevented by imoxin treatment. There were main effects of insulin and imoxin on the protein levels of p-IRS1 (S636/639, Figure 5C), although there were no differences in protein levels of p-IRS1 (S636/639) amongst all of the groups. On the other hand, palmitate significantly reduced insulin-stimulated IRS1 compared with BSA-control and there were main effects of palmitate, insulin, imoxin, palmitate*imoxin and palmitate*imoxin*INS in protein levels of IRS1 (Figure 5D). However, there were no differences among all of the groups and no main effects found in IRS1 phosphorylation at Ser636/639 normalized to total IRS1 (Figure 5E). In addition to p-IRβ and IRS1, palmitate significantly lowered protein levels of p-Akt (S473) and p-AS160 and decreased Akt phosphorylation (p-Akt/Akt), which were augmented by imoxin treatment (Figure 6). There was a main effect of insulin, resulting in lower total Akt across all condition, but no main effect of palmitate or imoxin, and no interactions. Importantly, differences in total Akt did impact the interpretation of Akt phosphorylation. No differences in total AS160 were observed across conditions.

## Discussion

This study investigated the beneficial effects of imoxin on saturated fatty acid-induced ER stress and insulin resistance in skeletal muscle myotubes. Our results demonstrate the ameliorative role of imoxin on palmitate-induced ER stress and on palmitate-induced suppression of the insulin signaling pathway. The current study found an elevation of PKR phosphorylation at Thr446 in the presence of palmitate which was inhibited imoxin treatment (Figure 1). In addition, imoxin suppressed palmitate-induced protein expression of molecular markers of ER stress such as GRP78, CHOP, ATF6, and XBP-1s (Figure 2). Moreover, we found that imoxin significantly reduced palmitate-induced phosphorylation of JNK in myotubes (Figure 3). Lastly, imoxin promoted insulin signaling and glucose uptake suppressed by palmitate (Figures 4–6).

Elevated blood level of FFAs is a signature in obesity and plays in crucial role in insulin resistance and inflammation in the insulin target tissues such as liver, adipose tissue, and skeletal muscle (Boden, 1998). Palmitate is the most abundant saturated FA and is responsible for 20%–30% of total fatty acids in the human body (Carta et al., 2017). Among the major insulin target tissues, skeletal muscle is a key tissue because skeletal muscle is responsible for up to 80% of insulin-mediated glucose utilization under normal physiological status (Li et al., 2018). Therefore, FFA-induced insulin resistance in skeletal muscle is strongly associated with the development of type 2 diabetes (T2DM) in obesity. In addition to insulin resistance, FFA can induce ER stress in skeletal muscle (Perry et al., 2018). Interestingly, ER stress itself can promote insulin resistance through activation of lipogenic and/or gluconeogenic gene expression (Lee et al., 2008; Ning et al., 2011; Flamment et al., 2012). Furthermore, it has been shown that ER stress can directly disrupt insulin receptor signaling mostly through the IRE1 pathway (Ning et al., 2011). Activation of the IRE1 transmembrane sensor results in JNK phosphorylation, which directly mediates phosphorylation of insulin receptor substrate 1/2 (IRS1/2) and blocks insulin signaling (Hilder et al., 2003; Flamment et al., 2012; Peng and He, 2018). Hence, to stop the development of T2DM in obesity, it is required to explore proper therapeutics to break the vicious cycle of ER stress and insulin resistance induced by saturated FAs such as palmitate.

PKR is a serine/threonine kinase which was originally identified as a pathogen sensing molecule (Nakamura et al., 2010). Later, it was elucidated that PKR is involved in inflammation and whole-body metabolism (Gal-Ben-Ari et al., 2018). Imoxin is a drug that inhibits action of PKR by blocking the ATP-binding site of PKR and consequently preventing its phosphorylation and activation (Hage Hassan et al., 2016). In a screen of 26 promising small molecules, imoxin was identified as the highest affinity inhibitor of PKR (Jammi et al., 2003). Despite this specificity, the potential for off-target effects of imoxin cannot be ruled out. For example, imoxin has been shown to inhibit CDK activity in neurons, eliciting therapeutic effects independent of PKR signaling (Chen et al., 2008). Thus, it cannot be concluded with certainty that all of the effects observed were directly through PKR inhibition. Nonetheless, PKR phosphorylation was clearly suppressed by imoxin, concomitant with reductions in ER stress and improvements in insulin signaling and glucose uptake.

Previously, our group observed that imoxin prevented glucocorticoid-induced elevation of muscle-specific E3 ubiquitin ligases and subsequent atrophy (Eo et al., 2020). Interestingly, tunicamycin significantly promoted PKR phosphorylation at Thr446 which was prevented by imoxin treatment (Eo and Valentine, 2021). Hage Hassan and others reported that low-grade but long002Dterm palmitate exposure promoted PKR mRNA expression as well as PKR phosphorylation at Thr451 in C2C12 myotubes (Hage Hassan et al., 2016). Like the previous studies, palmitate significantly increased PKR phosphorylation at Thr446 whereas imoxin ameliorated palmitate-induced PKR phosphorylation (Figure 1). Our result is in accordance with Nakamura et al.‘s study reporting that palmitate increased PKR activity in primary mouse embryonic fibroblasts (MEFs) (Nakamura et al., 2010). At the same time, Nakamura et al. demonstrated that PKR depletion prevented action of thapsigargin which is a widely used ER stress inducer (Nakamura et al., 2010). Likewise, ER stress associated biomarkers were evaluated and molecules involving in insulin signaling pathway were examined in the current study.

Active PKR phosphorylates eIF2α and p-eIF2α selectively promotes Cap-independent translation of some proteins including ATF4 and CHOP which involve in UPR (Mccormick and Khaperskyy, 2017; Tang et al., 2019). This implies that PKR may play a crucial role in ER stress. Indeed, small-molecule PKR inhibitors including imoxin and 2-aminopurine (2-AP) suppressed tunicamycin-mediated ER stress in pancreatic β-cell (Yalcin et al., 2020). Additionally, PKR-knockdown showed protective effects against ER stress and ER stress-induced apoptosis in human embryonic kidney cells (Lee et al., 2007). In our previous study, imoxin prevented tunicamycin-induced ER stress in C2C12 myotubes (Eo and Valentine, 2021). In the current study, it was evaluated whether imoxin treatment can suppress palmitate-induced ER stress in C2C12 myotubes, a physiologically relevant inducer of ER stress and insulin resistance. Several studies have shown that palmitate administration at 0.5 mM concentration for 6–24 h increases ER stress in skeletal muscle cell (Woodworth-Hobbs et al., 2017; Li et al., 2018; Perry et al., 2018). The current study demonstrated that palmitate increase protein levels of GRP78 and CHOP which were significantly lowered by imoxin treatment (Figure 2B). Upon ER stress, GRP78 is dissociated from ER membrane sensors including PERK, IRE1 and ATF6 and then binds to unfolded/misfolded proteins due to its affinity (Guzel et al., 2017). It was reported that short-term treatment with ER stress inducers increases the gene expression of GRP78 up to 25 fold (Ibrahim et al., 2019). Moreover, CHOP is downstream of all the ER stress branches and is known as a key player in ER stress-mediated apoptosis (Hu et al., 2018).

In addition to GRP78 and CHOP, the current study showed that palmitate increased protein levels of ATF6 and XBP-1s and PERK phosphorylation which were all prevented by imoxin treatment (Figures 2C,D). The results imply that effect of imoxin encompasses all three UPR branches against saturated FA. Specifically, XBP-1 splicing results from IRE1 activation (Afroze and Kumar, 2019). IRE1 can activate the apoptotic-signaling kinase-1 (ASK1) and its downstream kinases such as JNK (Hu et al., 2018). Despite the robust inhibitory effects of imoxin against palmitate-induced PERK phosphorylation, imoxin had no effect on ATF4 protein content. The cause of discrepancy is unclear, but could involve timing, in which imoxin did not fully inhibit downstream signaling, or that negative feedback was already received. Another possibility is that the analysis here was limited to whole cell lysate, whereas nuclear translocation of ATF4 is required for its transcriptional effects. Nonetheless, the palmitate-induced increase in CHOP, one of the downstream targets of ATF4, was clearly prevented by imoxin.

As mentioned above, JNK activation leads to IRS1 degradation and reduces cellular insulin sensitivity. Therefore, JNK is a key link between ER stress and insulin resistance. The current study found that palmitate induced JNK activation which was suppressed by imoxin treatment (Figure 3), suggesting that imoxin can attenuate palmitate-induced ER stress and impairments in insulin signaling in skeletal muscle cells. However, the current data are limited to p-JNK, in the absence of total JNK, which may limit this interpretation. In addition, p-JNK was significantly reduced by imoxin treatment alone without palmitate treatment. This might be due to the direct interaction between imoxin and JNK (Sud et al., 2016; Jiang et al., 2020). Therefore, palmitate appears to induce JNK activation, which is likely suppressed by imoxin, potentially through direct and indirect interactions between imoxin and JNK.

It was reported that PKR-deficiency protected mice from lipid infusion-induced insulin resistance and augmented whole-body glucose disposal rates (Nakamura et al., 2010). In particular, the study demonstrated that PKR deficient (PKR^−/−^) mice showed higher glucose uptake in gastrocnemius muscle compared to PKR^+/+^ mice (Nakamura et al., 2010). In the current study, imoxin significantly augmented glucose uptake which was decreased by palmitate treatment (Figure 4). This outcome is further supported by our data showing that imoxin promoted the insulin signaling pathway suppressed by saturated FA.

In response to elevated concentrations, insulin binds with IR and its β-subunits are phosphorylated (Olivares-Reyes et al., 2009). p-IRβ recruits IRS1 which serve as a docking protein for mitogen-activated protein kinases (MAPKs) and phosphatidylinositol 3-kinase (PI3K)/Akt (Olivares-Reyes et al., 2009). Akt is a key molecule which is responsible for the metabolic actions of insulin including glucose uptake (Olivares-Reyes et al., 2009). When Akt is phosphorylated at Ser473 and/or Thr308 and activated, p-Akt phosphorylates AS160, which results in translocation of glucose transporter 4 (GLUT4) storage vesicle (Jiang et al., 2008; Olivares-Reyes et al., 2009).

Saturated FAs are well-known factors in the impairments of insulin sensitivity in obesity (Kennedy et al., 2009). Several studies have shown that palmitate blocks activation of insulin receptor signaling (Chavez and Summers, 2003; Reynoso et al., 2003; Kennedy et al., 2009). In addition, saturated FA can directly cause skeletal muscle inflammation which worsens myopathies and metabolic disorders (Kennedy et al., 2009). Many researches have shown that palmitate increased expression and secretion of inflammatory cytokines such as interleukin-6 (IL-6) and tumor necrosis factor *α* (TNFα) and stimulates nuclear factor kappa B (NF-κB) signaling pathway in skeletal muscle (Sinha et al., 2004; Weigert et al., 2004; Jove et al., 2005; Jove et al., 2006).

Saturated FA-induced inflammation can be found not only in skeletal muscle but also many metabolic organs including liver and adipose tissue (Silva Figueiredo et al., 2017; Charles-Messance et al., 2020). For this reason, chronic low-grade inflammation in obesity, called metaflammation, is strongly associated with insulin resistance and the development of T2DM (Chen et al., 2015; Khodabandehloo et al., 2016). In a previous study, PKR inhibition or depletion significantly suppressed expression of inflammatory cytokines such as IL-6 and TNFα in white adipose tissue of ob/ob mice (Nakamura et al., 2014). Moreover, a research group demonstrated that PKR is involved in the activation of NOD-like receptor (NLR) family pyrin domain-containing 3 (NLRP3) inflammasome and further the maturation of IL-1β (Lu et al., 2012). Likewise, our group reported that imoxin attenuated endotoxin-induced inflammation in mouse skeletal muscle (Valentine et al., 2018). These studies suggest PKR inhibition as a potential therapeutic against insulin resistance as well as inflammation in obesity. In our previous study, imoxin promoted insulin sensitivity by upregulation of IRS1 phosphorylation which was suppressed by tunicamycin, one of the ER stress inducers and the pattern or IRS1 phosphorylation followed by IRβ phosphorylation which is the first step of insulin receptor signaling (Eo and Valentine, 2021). However, there were no changes in IRS1 phosphorylation in the imoxin treatment in the current study. This might be due to the difference in complex mode of action and between tunicamycin and palmitate. Nevertheless, in the current study, the results showed that palmitate treatment significantly suppressed insulin signaling cascade by reducing protein levels of p-IRβ and p-AS160 and Akt phosphorylation in response to insulin in C2C12 myotubes. However, imoxin prevented the palmitate-induced reduction in all three proteins (Figures 5, 6) and promoted glucose uptake in response to insulin treatment (Figure 4), which appears to be independent of IRS1. Collectively, imoxin treatment significantly preserved insulin sensitivity and glucose uptake against saturated FA in skeletal muscle cells. Even though the current study demonstrated a protective effect of imoxin on insulin resistance in myotubes, it is still unclear whether the impact of imoxin on ER stress and insulin resistance is specifically mediated through PKR inhibition. Therefore, further study would be required to demonstrate the specificity of imoxin in PKR inhibition against ER stress and insulin resistance.

In conclusion, the current results provide evidence that palmitate increased PKR phosphorylation at Thr446 in skeletal muscle cells, which was inhibited by imoxin treatment. In addition, imoxin prevented palmitate-induced excessive UPR pathways in myotubes. Furthermore, imoxin ameliorated impairments in insulin signaling and glucose uptake interfered by palmitate. These findings imply the therapeutic potential of imoxin for the improvement of skeletal muscle ER stress and insulin resistance in individuals with obesity.

## Data Availability

The original contributions presented in the study are included in the article. Further inquiries can be directed to the corresponding author.
